# The Polyoma Virus Large T Binding Protein p150 Is a Transcriptional Repressor of c-MYC

**DOI:** 10.1371/journal.pone.0046486

**Published:** 2012-09-28

**Authors:** Chang Kyoo Sung, Hyungshin Yim, Hongcang Gu, Dawei Li, Erik Andrews, Sekhar Duraisamy, Cheng Li, Ronny Drapkin, Thomas Benjamin

**Affiliations:** 1 Department of Microbiology and Immunobiology, Harvard Medical School, Boston, Massachusetts, United States of America; 2 Dana Farber Cancer Institute, Department of Medical Oncology, Center for Molecular Oncologic Pathology, Boston, Massachusetts, United States of America; 3 Dana Farber Cancer Institute, Department of Biostatistics and Computational Biology, Boston, Massachusetts, United States of America; 4 Brigham and Women's Hospital, Department of Pathology, Boston, Massachusetts, United States of America; Wayne State University, United States of America

## Abstract

p150, product of the SALL2 gene, is a binding partner of the polyoma virus large T antigen and a putative tumor suppressor. p150 binds to the nuclease hypersensitive element of the c-MYC promoter and represses c-MYC transcription. Overexpression of p150 in human ovarian surface epithelial cells leads to decreased expression, and downregulation to increased expression, of c-MYC. c-MYC is repressed upon restoration of p150 to ovarian carcinoma cells. Induction of apoptosis by etoposide results in recruitment of p150 to the c-MYC promoter and to repression of c-MYC. Analysis of data in The Cancer Genome Atlas shows negative correlations between SALL2 and c-MYC expression in four common solid tumor types.

## Introduction

Oncogenic DNA viruses target tumor suppressor genes of their hosts for inactivation ostensibly to promote cell cycle progression and to block apoptosis as essential steps in virus replication. These functions are also critical for cell transformation and tumor induction by these viruses [1]. The highly oncogenic mouse polyoma virus (Py) stands apart from SV40 and other DNA tumor viruses in failing to target p53 for inactivation or destruction. A screen was previously designed to identify tumor suppressors or other host factors with which Py must interact in order to replicate. The product of the SALL2 gene (p150) was identified as a Py large T binding protein using this screen [2]. p150 binds at the C-terminus of Py large T in a region bearing no homology to SV40 large T. p150 blocks Py DNA replication and binding of p150 by Py large T overcomes this inhibition. A Py mutant unable to bind p150 is unable to replicate and fails to induce a broad spectrum of tumors in the mouse [2]. Targeting of p150 by the human papilloma virus HPV16 E6 oncoprotein has recently been reported [3].

Evolutionarily conserved SALL genes encode multi-zinc finger transcription factors that function in embryonic development in invertebrate and vertebrate species [4] including man [5], [6], [7], [8]. Unique within this family, SALL2 has been implicated as a possible tumor suppressor [9], [10]. SALL2 has growth arrest and proapoptotic functions that overlap with those of p53. Specifically, p150 binds to the p21^Cip1/Waf1^ promoter in regions adjacent to the known p53 binding sites and transactivates p21^Cip1/Waf1^ in the absence of p53 [9]. p150 also binds to the BAX promoter and activates transcription [9], [11]. Restoration of p150 to human ovarian carcinoma cells deficient in p150 expression results in partial inhibition of tumor growth in SCID mice accompanied by elevated levels of p21^Cip1/Waf1^ and BAX [9]. SALL2 has also been recognized and characterized as a ‘quiescence factor’, essential for arresting growth of human fibroblasts under conditions of serum deprivation [12]. When treated with SALL2-siRNA, serum-deprived cells fail to arrest [12]. Upon restoration of serum, p150 is rapidly degraded as cells re-enter the cell cycle. Factors that regulate SALL2 expression at the transcriptional and posttranslational levels as a function of growth conditions have been identified [13].

The DNA sequence specificity of binding by p150 has recently been investigated [11]. The optimal consensus sequence for binding *in vitro* is the heptanucleotide GGG(T/C)GGG. p150 binds to GC-rich elements related to this sequence present in the promoter regions of p21^Cip1/Waf1^ and BAX [11]. The present investigation was undertaken to determine whether the protooncogene c-MYC may come under negative regulation by p150, consistent with the frequent overexpression of c-MYC in many forms of cancer and with the action of p150 as a putative tumor suppressor.

## Materials and Methods

### Cells and transfections

HOSE cells were telomerase-immortalized, HPV E6-transformed human ovarian surface epithelial cells [14], [15]. Ovarian carcinoma-derived RMUGS cells which are deficient in p150 [9] were obtained from the American Type Culture Collection. Cells were grown in DMEM with 10% fetal bovine serum. pcDNA 3.1 (Invitrogen) and pcDNA3.1-p150 were transfected with lipofectamine 2000 (Invitrogen) into HOSE cells for 48 hours. Negative control siRNA (Invitrogen, #45-2001) and SALL2 siRNA (Invitrogen # HSS 109406) were transfected with oligofectamine (Invitrogen) for 48 hours.

### Antisera

Polyclonal antisera were raised in rabbits against the N-terminus (amino acids 1–550) and the C-terminus (amino acids 717–1005) of human p150 purified as GST fusions [11]. Antisera were purified by flow through over a GST column and binding to Staph A agarose beads. c-MYC epitope antibody was from Santa Cruz ; it recognizes the 62 kDa c-Myc2 protein referred to as c-MYC in this study. The anti-GAPDH was from Calbiochem.

### Western blots

Immunoblotting was carried out on cell extracts using an Odyssey infrared imaging system (LI-COR Biosciences, Lincoln, NE). Intensity values were determined with LI-COR Odyssey software (Li-COR Biosciences).

### Chromatin immunoprecipitation (ChIP)

ChIP was performed as described [16] with slight modifications. For binding of endogenous p150 to the c-MYC promoter in HOSE cells, Staph A-agarose beads coated with N- or C-terminal p150 antibody or normal rabbit sera were used. Immunoselected chromatin fragments were subjected to PCR (32 cycles) with a primer set (5′-CTCTCTTACTCTGTTTACATCCTAG and 5′-CTGGAATTACTACAGCGAGTTAG) for c-MYC amplification. Band intensities of the amplified DNA were revealed on a 1% agarose gel. ChIP assays were performed on HOSE cells with or without prior si-RNA SALL2 knockdown. Control- or SALL2-siRNA was introduced into HOSE cells. At 48 hours post transfection, sonicated chromatin was immunoprecipitated with N-terminal p150 antibody. qRT-PCR assays were performed with primer sets (c-MYC promoter, 5′-GCGTAGTTAATTCATGCGGCTCTC and 5′- CCCACGCCCTCTGCTTTGGGAACC; aldolase A promoter, 5′- CGGTCTGTTCGTTGCACAGAGTAG and 5′-GTTGAGGCAGTAGACAGAGAAAGC). The threshold values of each sample were normalized with those of corresponding 1% input control sample. To determine the effects of etoposide on p150-binding to the c-MYC promoter, HOSE cells were transfected with p150 expression vector for 48 hours and incubated for an additional 22 hours with etoposide (40 µM ) to induce apoptosis. ChIP was carried out with anti-N-terminal p150 antibody. Immunopurified chromatin fragments were amplified by RT-PCR.

### Quantitative RT-PCR

Total RNA was isolated using RNeasy® kit (Qiagen), reversed transcribed using QuantiTect Reverse Transcription Kit (Qiagen) and quantitated by RT-PCR using specific primers: SALL2 F: 5′-CACGAATCCGAGAGGAGCTCTC, R: 5′-CACCATTACAGGAGGGTCAGTAG; c-Myc F: 5′-GCTCCACCTCCAGCTTGTAC, R: 5′CGAGCTGCTGTCGTTGAGAG; Aldolase A F: 5′-CGCAGAAGGGGTCCTGGTGA, R: 5′-CAGCTCCTTCTTCTGCTCCGGGGT. qRT-PCR was carried out on a Roche LightCycler 480 using SYBR Green Master Mix. The data were analyzed by the comparative C_T_ (ΔΔC_T_) method and quantitated relative to the aldolase A gene and normalized to the controls.

### Gel shift assays for binding of p150 to the c-MYC promoter

Oligonucleotides containing the NHE III_1_ of the human c-MYC promoter (5′-TGGGGAGGGTGGGGAGGGTGGGGAAGG), the p150 consensus binding sequence (CS) (5′-GGATCACTGGGTGGGAATCACGCT) and Oct1 binding site (5′-TGTCGAATGCAAATCACTAGAA) and their complementary sequences were purchased from IDT (Coralville, Iowa). Annealed complementary fragments were radiolabeled with [γ-^32^P] dATP. Binding reactions were carried out in the presence of 100 ng purified GST or GST tagged p150 in reaction buffer containing 10 mM HEPES (pH 7.5), 25 nM KCl, 2.5 mM MgCl, 5 µl ZnCl, 3% glycerol, 2 µg BSA and 200 ng Poly(dA-dT). For specific and nonspecific competition, 50 molar excess unlabeled oligonucleotides were added to the mixture.

### Luciferase reporter assay

Reporter plasmids myc-Luc and myc-Luc-ΔNHE with and without the NHE III_1_ respectively were constructed using two primer sets: myc-Luc, 5′-ATAGATCTCTCTTACTCTGTTTACATCCTAGAGC and 5′-ATAAGCTTCCGGGAGGGGCGCTTATGGGGAGGG; myc-Luc-ΔNHE, 5′- ATAAGCTTCCTCAGCCGTCCAGACCCTCGCATT and 5′- ATAAGCTTGGAGACTCAGCCGGGCAGCCGAGCAC. Amplified fragments were cloned into pGL3 and the constructs verified by sequencing. The vectors myc-Luc and myc-Luc-ΔNHE and pRL-TK were introduced into HOSE cells pretreated with control siRNA or SALL2-siRNA oligonucleotides. For a dose-dependent reporter assay, RMUGS cells were transfected with myc-Luc, pRL-CMV and pcDNA or pcDNA-p150. After 48 hours of incubation at 37°C, the transfected cells were subjected to luciferase activity assays (Dual-Luciferase Reporter System; Promega, Madison, WI). Firefly luciferase values were normalized to those of Renilla luciferase.

### Correlations between SALL2 and c-MYC expression levels in tumors

Gene expression data from The Cancer Genome Atlas (TCGA) were accessed and downloaded from TCGA Data Portal (https://tcga-data.nci.nih.gov/tcga/). Cancer types selected for correlation analysis were those having more than 100 samples with Level 3 Expression-Gene data (https://tcga-data.nci.nih.gov/tcga/tcgaDataType.jsp) and ones known to arise from tissues that normally express SALL2 based on data in the mouse [2] and human ovarian epithelial cells [9]. This resulted in selection of ovarian serous cystadenocarcinoma (OVCA), glioblastoma multiforme (GBM), lung squamous cell carcinoma (LUSC) and breast invasive carcinoma (BRCA). For all data sets, tumor specimen-specific SALL2 and MYC expression levels were retrieved, compiled, and entered into the SAS JMP Pro 9.0.0 software package. Scatter plots of SALL2 by MYC were generated using the software's “Fit Y by X” command. Pearson correlation coefficients and associated p values were calculated using the default Restricted Maximum Likelihood (REML) method.

## Results and Discussion

### p150 binds to the NHE III_1_ of the c-MYC promoter

**Figure 1:**
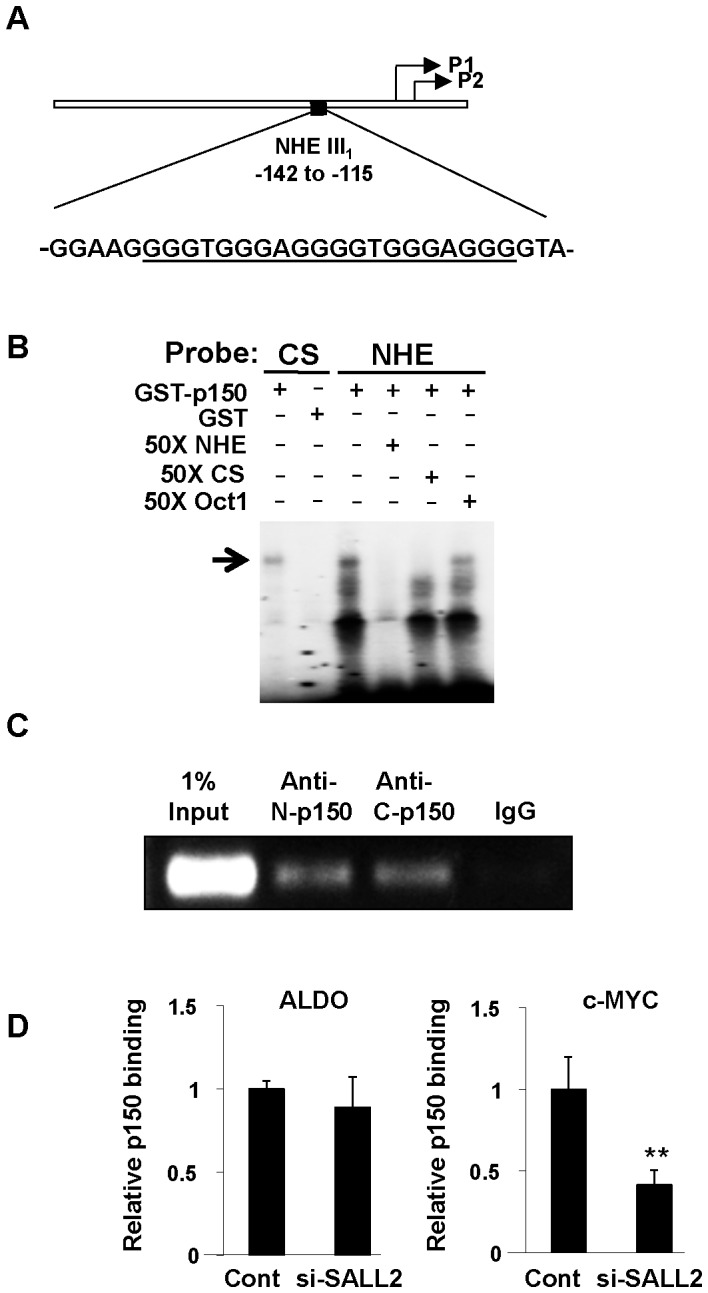
GST-p150 binds to the c-MYC promoter region *in vitro* and *in vivo*. (A) The GC-rich nuclease hypersensitive element NHE III_1_ of the human c-MYC promoter. Consensus binding sites for p150 are underlined. Approximate positions of transcriptional start sites are shown (P1, P2). (B) An electrophoretic mobility shift assay shows binding of GST-p150 to the labeled NHE III_1_ and to the consensus binding sequence (CS) GGGTGGG- as control (<$>\raster(75%)="rg1"<$>). 50× molar excess of unlabeled NHE III_1_ and CS compete for binding while the unrelated Oct1 olionucleotide does not. (C) Chromatin immunoprecipitation assays with antibodies to the N- and C-terminal portions of p150 show binding of endogenous p150 to the NHE III_1_ region of the c-MYC promoter in HOSE cells. (D) SALL2-knockdown leads to reduced binding of p150 to the c-MYC promoter. HOSE cells were treated with SALL2 siRNA or control oligonucleotide and followed by chromatin immunoprecipitation and quantitative RT-PCR assays. The threshold cycle values of immunoprecipitates were normalized to those of 1% input control samples. The human aldolase A promoter region was included as a negative control. Estimates of binding were based on triplicate samples. ** denotes p<0.01 based on Student *t* test run on HOSE cells treated with SALL2-siRNA or control RNA.

Several approaches have been used to determine whether p150 binds to the c-MYC promoter region and regulates its expression. Initial focus was on the GC-rich nuclease-hypersensitive element (NHE III_1_) as a key regulator of c-MYC expression. NHE III_1_ has tandem repeats of the optimal consensus binding sequence established for p150 [11] (Figure 1A). The NHE III_1_ can adopt a four stranded ‘quadruplex’ secondary structure known to be targeted by zinc-finger transcription factors and to be associated with silencing of c-MYC transcription [17], [18]. A recombinant GST fusion protein (GST-p150) was expressed and purified from 293 cells [11]. GST-p150 was used in a gel shift assay with radioactive NHE III_1_ (Figure 1B). GST-p150 clearly binds to the NHE III_1_ as well as to the consensus sequence (CS) oligonucleotide as a control. A 50-fold molar excess of unlabeled NHE III_1_ or CS oligonucleotide competed for binding by the labeled NHE III_1_ while the unrelated Oct1 oligonucleotide did not.

To determine whether p150 binds to the c-MYC promoter *in vivo*, chromatin immunoprecipitation was carried out using established human ovarian surface epithelial cells (HOSE). Binding of endogenous p150 to a region of the c-MYC promoter encompassing the NHE III_1_ was evident using either of two polyclonal antibodies to the human protein (Figure 1C). SALL2 knockdown resulted in a decreased level of p150 binding to the c-MYC promoter region (Figure 1D).

### p150 negatively regulates c-MYC expression

**Figure 2:**
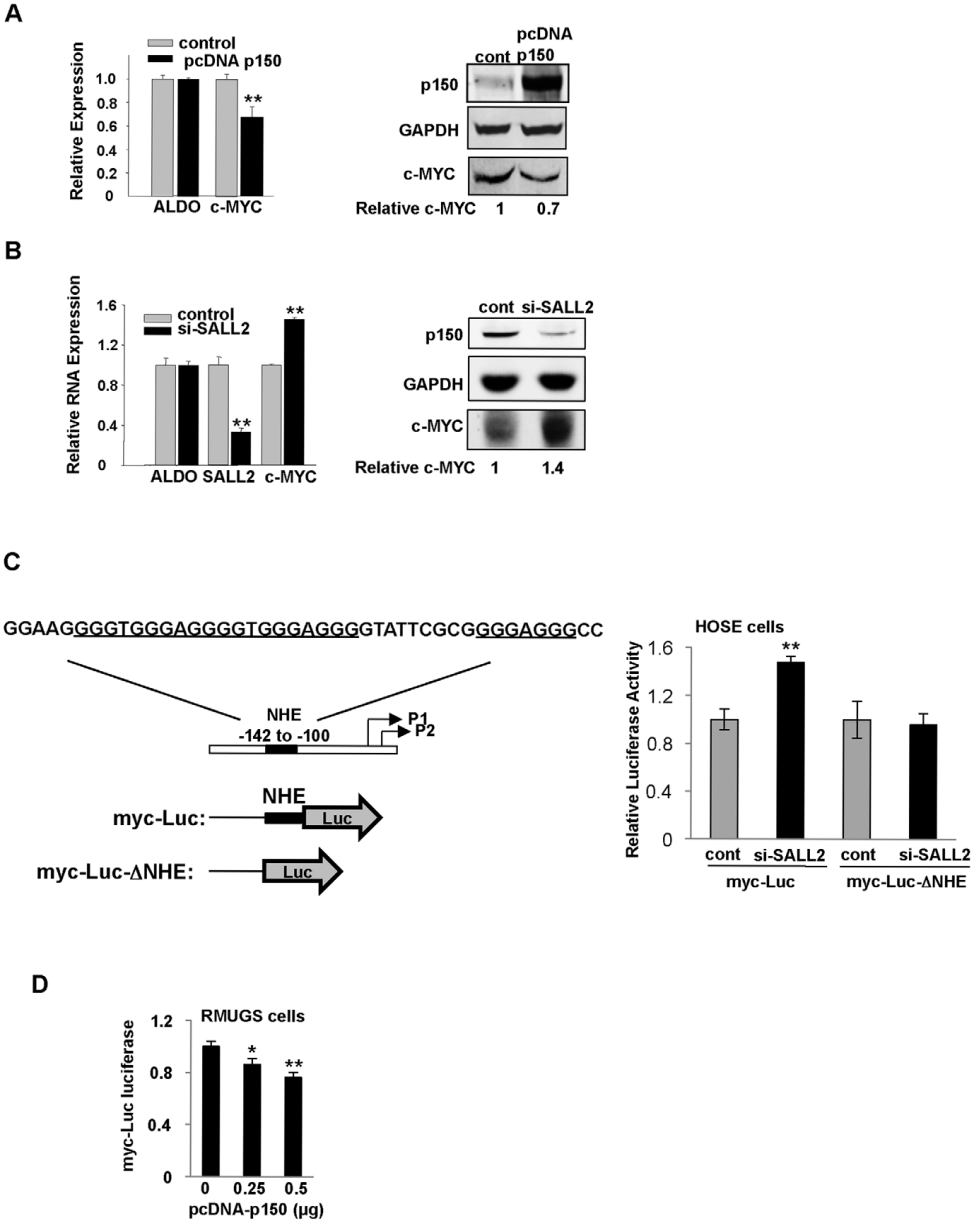
p150 represses c-MYC expression. (A) Left - overexpression of p150 results in reduced c-MYC expression in HOSE cells shown by quantitative RT PCR. Right – immunoblotting for c-MYC. (B) Left - siRNA-knockdown of endogenous SALL2 in HOSE cells leads to increased c-MYC expression by quantitative RT PCR. Right - immunoblotting for c-MYC. (C) Left - Human c-MYC promoter region and luciferase reporters used in promoter activity assays. The NHE region (−142 to −100) including p150 consensus binding sites (underlined) was deleted in the reporter myc-Luc-ΔNHE. Right - siRNA to SALL2 in HOSE cells results in increased expression of myc-Luc but has no effect on myc-Luc-ΔNHE. (D) Expression of exogenous p150 in p150-deficient RMUGS ovarian cancer cells leads to decreased expression of the reporter myc-Luc. All histograms are based on triplicate determinations. * and ** denote p<0.05 and p<0.01, respectively, comparing means of experimental and control.

To investigate whether binding of p150 to the c-MYC promoter results in activation or repression of c-MYC, levels of SALL2 expression were manipulated in HOSE cells and in RMUGS, an ovarian carcinoma-derived cell line lacking expression of p150 [9]. Overexpression of p150 in HOSE cells led to reduced levels of c-MYC RNA and a commensurate decrease in levels of the protein (Figure 2A). si-RNA targeting of SALL2 was previously shown to promote G1→S progression in these cells [9]. This treatment also led to increases in c-MYC mRNA (Figure 2B, left panel) and protein (Figure 2B, right panel).

HOSE cells pretreated with si-SALL2 or control RNA oligonucleotide were transfected with luciferase reporters with and without the NHE III_1_ (Figure 2C, left) and a pRL-TK-Renilla control vector for normalization. si-SALL2 RNA gave rise to increased expression of the NHE III_1_ reporter (Figure 2C, right). The ability of p150 to repress c-MYC expression was dependent on retention of the NHE III_1_ as the reporter plasmid lacking NHE III_1_ showed no response to si-SALL2 (Figure 2C, right). When RMUGS cells lacking p150 were transfected with increasing amounts of pcDNA- p150 expression vector, signals from the myc -Luc reporter were progressively reduced (Figure 2D).

These results demonstrate that p150 binds to the NHE III_1_ of the c-MYC promoter *in vitro* and *in vivo* and represses c-MYC transcription. The degree of transcriptional repression of c-MYC by p150 is relatively modest. Expression of c-MYC is tightly regulated, with small and often transient increases leading to cellular proliferation [19], [20]. Consistent with these results are earlier findings that downregulation of SALL2 stimulates growth of HOSE cells and that restoration of SALL2 in ovarian carcinoma cells results in suppression of tumor growth [9].

### An apoptotic signal leads to increased binding of p150 to the c-MYC promoter

**Figure 3:**
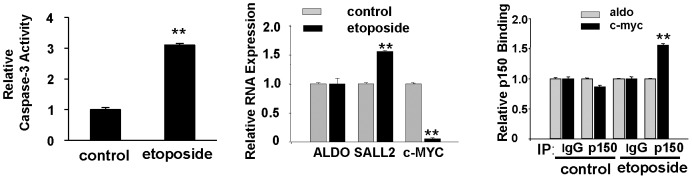
An apoptotic signal leads to increased binding of p150 to the c-MYC promoter. (A) Left - Etoposide-treated HOSE cells were monitored for apoptosis by caspase-3 activation. Middle - Quantitative RT-PCR shows a moderate increase in SALL2 and a large decrease in c-MYC expression following etoposide treatment. Right - ChIP followed by qRT-PCR shows increased binding of p150 to the c-MYC promoter in etoposide-treated cells. All histograms are based on triplicate determinations. ** denotes p<0.01 comparing means of experimental and control.

c-MYC can regulate apoptosis either positively or negatively depending on cell type and physiological conditions [21]. HOSE cells were treated with etoposide (40 µM for 22 hours) to induce apoptosis. A fluorimetric assay for caspase-3 was used to confirm the apoptotic response (Figure 3, left panel). Etoposide-treated cells were assayed for SALL2 and c-MYC mRNA. SALL2 was slightly induced and c-MYC strongly repressed following etoposide treatment (Figure 3, middle panel). The strong reduction in c-MYC RNA is most likely due in large part to p53 which is known to repress c-MYC [22], [23]. HOSE cells show a robust p53 response to etoposide (Figure S1). Chromatin immunoprecipitation with anti- p150 followed by qRT-PCR showed that etoposide treatment led to increased binding of p150 to the c-MYC promoter (Figure 3, right panel). Thus, recruitment of p150 to the c-MYC promoter occurs as part of a response to an apoptotic stimulus in HOSE cells. In other cell systems, modest increases in c-MYC expression lead to a proliferative response, while high levels of expression have been linked to tumor suppression and apoptosis [24], [25]. Negative regulation of c-MYC by SALL2 may function to maintain c-MYC levels within a normal range, avoiding thresholds that lead to overproliferation or apoptosis.

### SALL2 and c-MYC expression in tumors

**Figure 4:**
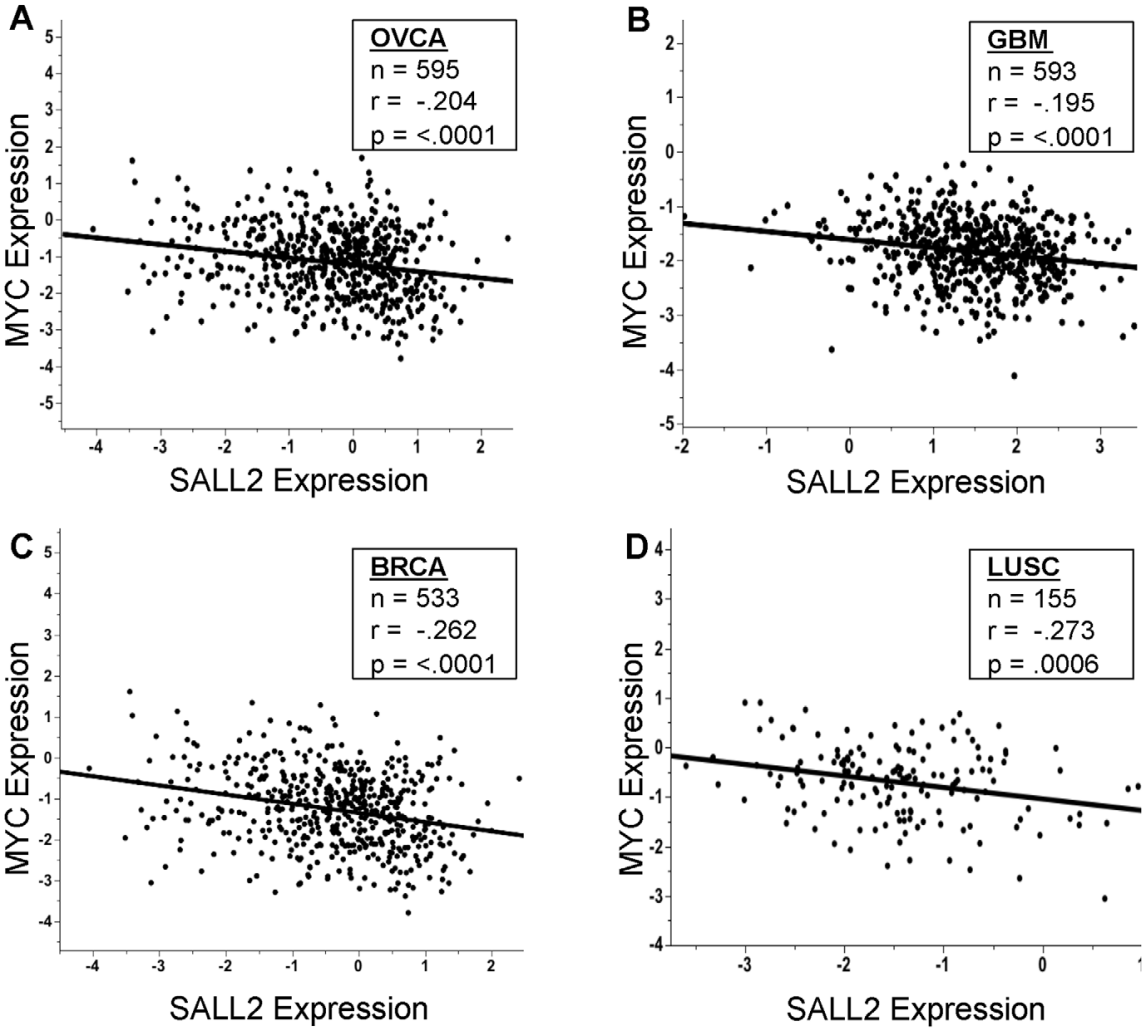
SALL2 by c-MYC expression scatterplots for cancer types in The Cancer Genome Atlas. (A) OVCA - ovarian serous cystadenocarcinoma. (B) GBM – glioblastoma multiforme. (C ) BRCA – breast invasive carcinoma. (D) LUSC – lung squamous cell carcinoma. n = number of samples; r = Pearson correlation coefficient; p = p-value of the correlation. The lines shown are those of best fit.

Overexpression of c-MYC due to increased transcription or DNA amplification accompanies the development of many forms of human cancer including ovarian [26], [27], [28], [29]. Transcriptional repression of c-MYC coupled with activation of p21^Cip1/Waf1^ and BAX by p150 constitute powerful growth inhibitory and proapoptotic functions consistent with action of SALL2 as a tumor suppressor. To determine whether overexpression of c-MYC is correlated with reduced expression of SALL2 in tumors, levels of SALL2 and c-MYC expression in The Cancer Genome Atlas were accessed. Four tumor types were selected for analysis based on their derivations from normal tissues known to express p150 in the mouse [2] and on high levels of expression in normal human ovarian surface epithelial cells (HOSE). These are ovarian serous cystadenocarcinoma (OVCA), glioblastoma multiforme (GBM), breast invasive carcinoma (BRCA), and lung squamous cell carcinoma (LUSC). These tumor types exhibit highly significant negative correlations between SALL2 and c-MYC expression. Inverse correlations were of similar magnitude, with Pearson coefficients (r) ranging from −.195 for GBM to −.273 for LUSC (Figure 4). Acute myeloid leukemia (AML) was analyzed as a control as there is currently no evidence for SALL2 expression in normal spleen [2] or other lymphoid tissue. For AML the negative correlation is not significant (r = −0.065, n = 197, p = .3677). Taken together, these results suggest that repression of c-MYC by SALL2 may contribute to a role of the latter in suppressing these forms of cancer. They point as well to the need for further understanding of the molecular functions of SALL2.

## Supporting Information

Figure S1 **Etoposide induces a strong p53 response in HOSE cells.** HOSE cells were treated with either DMSO or etoposide (40 µM) for 7 hours. Cells were lysed in PBS with 1% NP40, protease and phosphatase inhibitors and subjected to a western analysis. Anti-p150, GAPDH and phospho-specific p53 antibodies were used.
(TIF)
