# Supplementary figures and images for: The Polyoma Virus Large T Binding Protein p150 Is a Transcriptional Repressor of c-MYC
Source: PLoS One. 2012 Sep 28;7(9):e46486. doi: 10.1371/journal.pone.0046486 (PMC3460914; doi:10.1371/journal.pone.0046486)

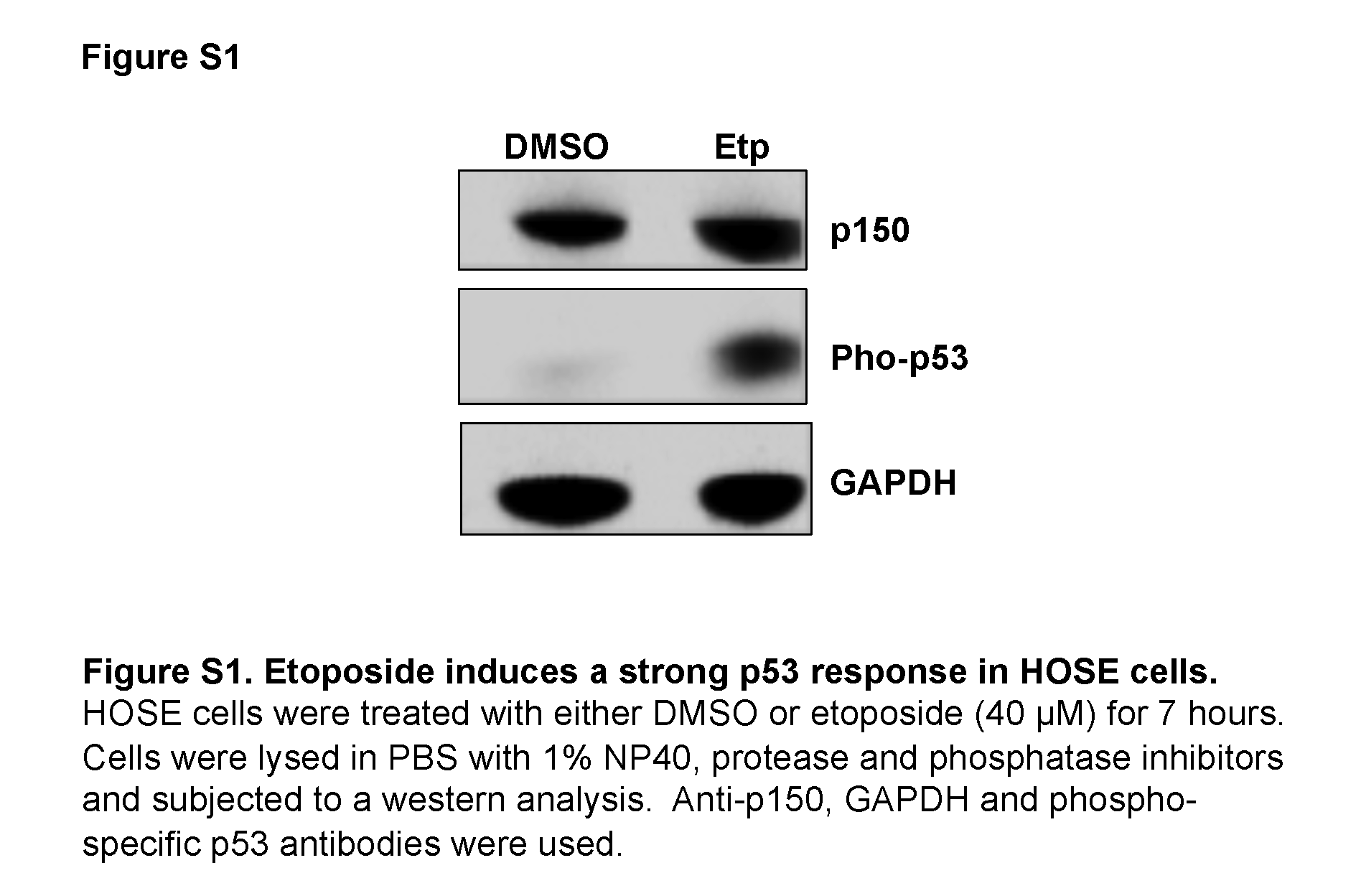

Supplement: Figure S1 — Etoposide induces a strong p53 response in HOSE cells. HOSE cells were treated with either DMSO or etoposide (40 µM) for 7 hours. Cells were lysed in PBS with 1% NP40, protease and phosphatase inhibitors and subjected to a western analysis. Anti-p150, GAPDH and phospho-specific p53 antibodies were used. (TIF) [file pone.0046486.s001.tif]
